# First‐In‐Human Application of Human Umbilical Cord‐Derived Extracellular Vesicles in Tethered Spinal Cord Release Surgery

**DOI:** 10.1002/jev2.70104

**Published:** 2025-06-19

**Authors:** Matthias Krause, Janina Gburek‐Augustat, Daniel Gräfe, Roman Metzger, Marco Ginzel, Christoph J. Griessenauer, Lukas Grassner, Daniel Weghuber, Johann Gradl, Daniela Auer, Tanja Schally, Stefan Rund, Carina Kals, Christina Folie, Elisabeth Bayer, Mario Gimona, Eva Rohde

**Affiliations:** ^1^ Department for Neurosurgery, Christian‐Doppler‐Klinik University Hospital Salzburg, Paracelsus Medical University Salzburg Austria; ^2^ Department of Pediatric Surgery University Hospital Salzburg, Paracelsus Medical University Salzburg Austria; ^3^ Department of Children's and Adolescence Health, Division of Neuropediatrics University Hospital Leipzig Leipzig Germany; ^4^ Department for Pediatric Radiology University Hospital Leipzig Leipzig Germany; ^5^ Department of Pediatrics University Hospital Salzburg, Paracelsus Medical University Salzburg Austria; ^6^ Department of Radiology University Hospital Salzburg, Paracelsus Medical University Salzburg Austria; ^7^ GMP Unit Paracelsus Medical University Salzburg Austria; ^8^ Department of Transfusion Medicine University Hospital Salzburg, Paracelsus Medical University Salzburg Austria; ^9^ Research Program “Nanovesicular Therapeutics” Paracelsus Medical University Salzburg Austria; ^10^ Ludwig Boltzmann Institute for Nanovesicular Precision Medicine at the Paris‐Lodron University Salzburg Salzburg Austria

**Keywords:** extracellular vesicle therapy, neuroprotection, Spina bifida, spinal bifida surgery, tissue regeneration

## Abstract

Spina bifida is a congenital neural tube defect that has a high risk of secondary neurological deterioration due to tethering of the spinal cord. We present the first application of human umbilical cord‐derived mesenchymal stromal cell‐derived extracellular vesicle (UC‐MSC‐EV) therapy in humans during spina bifida surgery. We discuss the application, post‐operative outcome and highlight the potential of extracellular vesicle therapy in the management of spina bifida.

Administration of extracellular vesicles containing therapeutically active agents has emerged as a potential new treatment modality for neurological disorders. By direct intrathecal application during surgery, UC‐MSC‐EVs can deliver therapeutic payloads to target cells and the extracellular environment, offering a novel approach to neuroprotection and tissue repair.

A 2‐year‐old girl diagnosed with spina bifida presented with progressive syringomyelia as sign of secondary tethered cord syndrome with intramedullary dermoid inclusion tumour after postnatal spina bifida repair. After pre‐operative assessment and multidisciplinary consultation, it was decided to proceed with spinal cord release surgery with the use of EV. During the surgical procedure, the tethered cord was released, dermoid and lipoma tissue were resected. Concurrently, UC‐MSC‐EVs were administered directly onto the released placode and spinal cord. Post‐operative MRI demonstrated a good de‐tethering effect and no medullary oedema. No adverse events were reported. The neurological deficit remained unchanged at 6 months follow‐up examination.

Intraoperative application of UC‐MSC‐EVs might be an option to ameliorate intrathecal scarring following spina bifida surgery. Whether EVs will result in significant effects for the long‐term neurological outcome needs to be studied in randomised clinical trials.

## 1 Introduction

Myelomeningoceles (MMC) and other open neural tube defects (ONTD) pose a significant risk for neurological deficits and secondary complications such as tethered cord syndrome. Advances in fetal surgery, particularly through in‐utero repair of spinal defects, have transformed treatment strategies over the past two decades. The landmark ‘Management of Myelomeningocele Study (MOMS)’ trial significantly influenced the clinical approach to the treatment of ONTD (Adzick et al. 2011). However, there is no specific treatment for the development of secondary tethered cord after spinal bifida repair.

So far, the sole treatment for tethered cord syndrome is by surgical release of the adherent neural structures from the surrounding scar tissue and dura, reducing sheer stress and stretching as well as allowing the spinal cord to float freely within the spinal canal. The procedure again harbours a risk of further damage to the neural structures as well as retethering by scar formation and intradural fibrosis (Almadori and Butler 2024; Borgstedt‐Bakke et al. 2020; Brock et al. 2019; Brun et al. 2023; Knerlich‐Lukoschus 2023). A critical role of inflammation in prenatal and postnatal damage and tethered cord syndrome in ONTD patients has been published recently by other authors (Chen et al. 2020; Cohrs et al. 2021, 2019; Knerlich‐Lukoschus 2023). These processes may play a crucial role for long‐term outcome of the ONTD patients. To date there is no satisfactory therapeutic modality to prevent or reverse intradural fibrosis (Almadori and Butler 2024; Borgstedt‐Bakke et al. 2020).

The effects of mesenchymal stromal cell‐derived extracellular vesicles (MSC‐EVs) on extracellular matrix turnover, local cell recruitment, proliferation, angiogenesis and fibrosis is well described (Davies et al. 2023; Huang and Yang 2021). Recent research has highlighted the potential of MSC‐EVs to modulate inflammation, fibrosis and tissue repair (Almadori and Butler 2024; Davies et al. 2023; Huang and Yang 2021). Thus, MSC‐EVs could offer a novel therapeutic approach to mitigate neurodegeneration after spina bifida surgery.

In this first‐in‐human case, we explore the feasibility of intraoperative application of human umbilical cord‐derived MSC‐EVs (UC‐MSC‐EVs) in a patient undergoing tethered cord release surgery, with the aim of reducing fibrosis and neurodegeneration.

## 2 Case Description

A 2‐year‐old female child with a prenatal diagnosis of ONTD was initially treated at the University Hospital Leipzig after birth. Postnatal spina bifida repair was performed microsurgically on the day of caesarean section. The girl required ventriculo‐peritoneal (VP)–Shunt implantation at the age of 6 weeks. The standard follow‐up protocol was offered, and the patient closely followed the recommended procedures according to the MOMS spina bifida study protocol and the recommendations of the American Spina Bifida Association for follow‐up protocols (Adzick et al. 2011; Borgstedt‐Bakke et al. 2020; Borthwick et al. 2013). Baseline MRI of the craniospinal axis have been performed at the age of 2 weeks.

**FIGURE 1:**
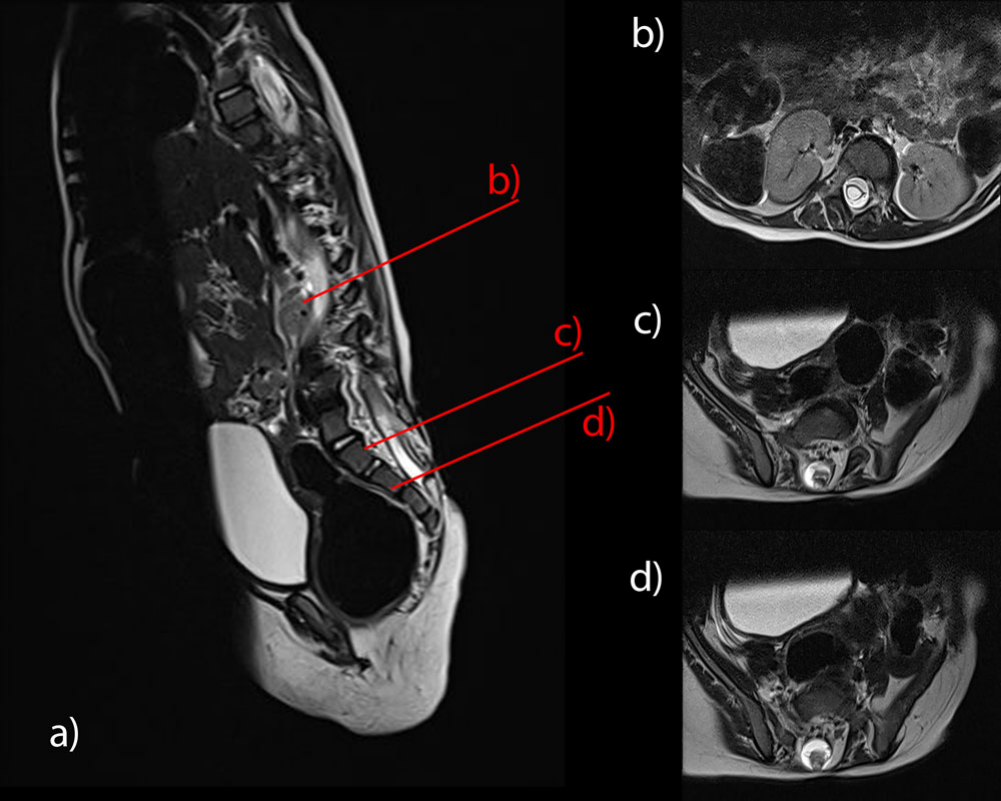
(a) Sagittal T2 MRI at age 1 year and corresponding axial T2 MRI slices, (b) at level of T11 with syringomyelia, (c) L5/S1 with dorsal lipoma and S1/2 with dermoid on the dorsal aspect of the placode with tethering.

At 1‐year routine follow‐up, MRI disclosed a progressive inclusion tumour at the site of the placode, tethered cord and syringomyelia within the lumbar and thoracic spinal cord (Figure 1a,b,d–f). Electrophysiological and neurological examinations did not show any deterioration of the motor functional level of L3 (modified Hoffer Scale H).

The radiological progressive alterations indicated a secondary tethered cord syndrome with intramedullary dermoid inclusion tumour after postnatal MMC repair. After thorough pre‐operative assessment and multidisciplinary consultation, the decision was made to proceed with spinal cord release surgery combined with EV therapy in the framework of an individual healing attempt. The parents were counselled by the principal investigators and the responsible surgeon before providing written informed consent for experimental intrathecal UC‐MSC‐EV application.

Due to the high risk of further neurological deterioration with life‐long severe consequences for the patient and family, ethical considerations also opted for the experimental treatment in light of the previous case‐based EV applications at our university hospital resulting in safe and preliminary successful clinical outcome. EVs have been produced in the certified manufacturing and procurement centre of PMU with a license to distribute for phases 1–3 clinical trials.

The child underwent tethered cord release in prone positioning with intraoperative neurophysiological monitoring (IONM) throughout the procedure until skin closure. IONM did not disclose any worsening with weak positive somatosensory‐evoked potential responses (SEP) at the lower limbs and motor‐evoked potential (MEP) responses in all motor segments, including sphincter MEP. Direct electromyography disclosed functionality of all motor nerve roots down to S2.

**FIGURE 2:**
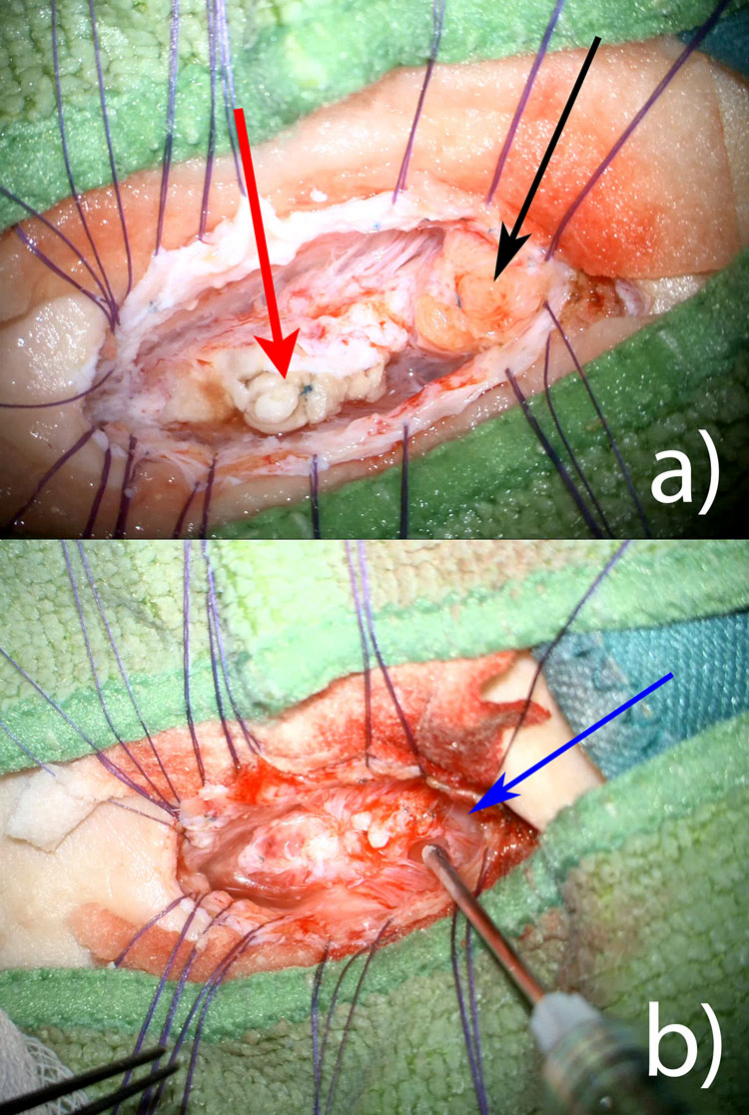
(a) Intraoperative microscopic view after dural opening with caudal intramedullary dermoid (left red arrow) and cranial dorsal lipoma (right black arrow). Note some of the sutures of primary postnatal surgery. (b) Intraoperative microscopic view after removal of the dermoid and lipoma. Syringomyelia (blue arrow) is also open. Application of UC‐MSC‐EVs with a syringe onto the surface.

Intraoperative inspection exposed a severe tethered cord with dermoid tissue inside the neural tube at the site of former neurulation. Adjacent to it, an intraspinal lipoma was present that also caused tethering to the dural sac (Figure 2a). After removal of both the dermoid and lipomatous tissue, 1.0 mL of UC‐MSC‐EVs containing fluid was applied to the open placode and into the syringomyelia canal (Figure 2b). Thereafter, neurulation was performed with 8‐0 prolene single sutures. Finally, 0.5 mL of UC‐MSC‐EVs were applied onto the sutured placode and the remaining 0.5 mL were filled into the dural sac at time of water tight closure. Thus, a total volume of 2.0 mL was applied intrathecally. No adverse events occurred during a post‐operative observation period of 6 months.

**FIGURE 3:**
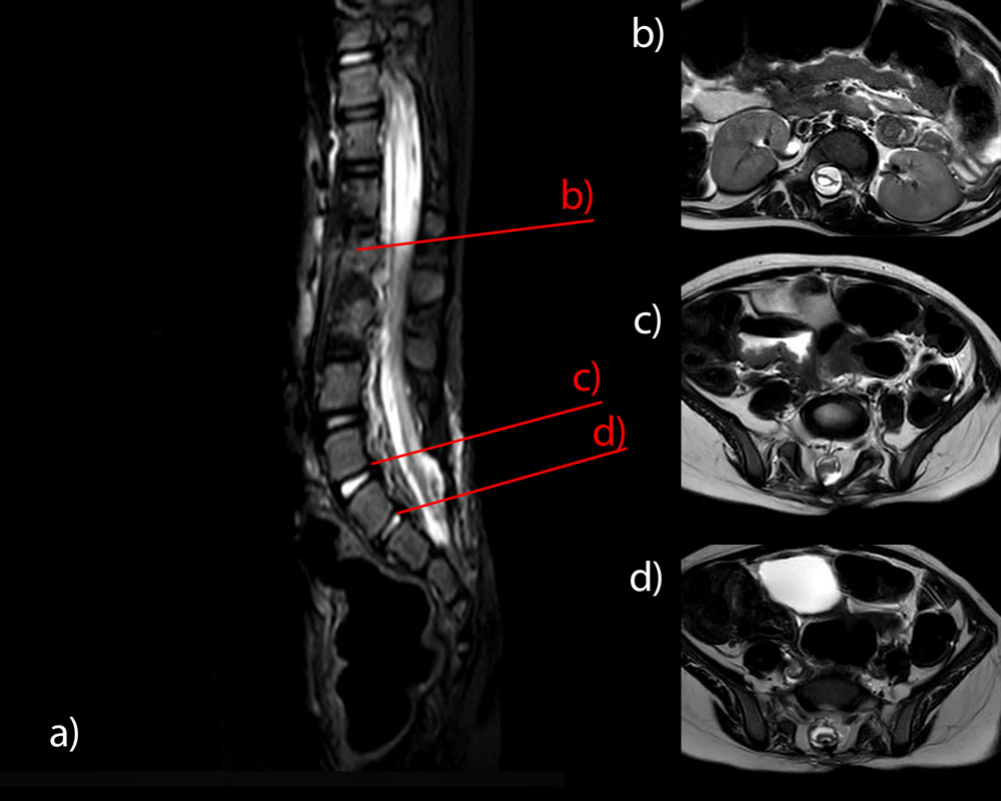
Post‐operative sagittal T2 (a) and corresponding Axial T2 MRI slices (b–d) at day 5: At level of T11 with reduced syringomyelia, L5 with removed lipoma and S1/2 with placode neurulation after dermoid removal with residual air inclusion.

The post‐operative course was uneventful, and the girl was discharged home after 7 days. The post‐operative MRI demonstrated a good de‐tethering effect, absence of post‐operative medullary oedema and diminished syringomyelia (Figure 3). Further neuropediatric follow‐up examinations 6 months after surgery did not disclose any adverse effects of the UC‐MSC‐EV application. The neurological status of the girl remained stable with slightly improved sensibility and temperature regulation in both legs. Motor function of the legs did not change and remained at functional level L3 (modified Hoffer Scale H).

### 2.1 Manufacturing and Characterisation of MSC and EV‐Containing Preparation

After primary isolation of umbilical‐cord (UC) derived MSCs from a single donor, cells were cultured in fibrinogen‐depleted culture medium in the presence of pooled human platelet lysate (HPL) at 5% CO_2_ and 37°C, as previously described (Warnecke et al. 2020; Martín‐Taboada et al. 2022). For the subsequent EV manufacturing process a Master (MCB) and a Working (WCB) cell bank from primary UC‐MSCs were established and tested according to European Pharmacopoeia for relevant phenotypic and genotypic markers by an external contractor (Virusure). Identity, viability, and purity of cells used for the production were ensured. Sterility testing, testing for the presence of mycoplasma, and determination of Endotoxin levels were performed by certified external laboratories. Full documentation has been provided by the contract laboratories and revealed compliance with all regulatory requirements. Generation and characterisation of cell banks was performed in accordance to the principles of ICH Q5D.

At the time of harvest of the EV‐containing conditioned medium from the WCB, the MSCs are at passage 8, which corresponds to a total of 17 population doublings and results in a sufficient number of producer cells for the clinical grade UC‐MSC‐EV suspension. No xenogenic substances were employed throughout the entire manufacturing process. MSCs were expanded in low glucose α‐MEM (Sigma‐Aldrich) supplemented with Dipeptiven (5.5 mg/mL, Fresenius‐Kabi, Graz, Austria) and 5% vol/vol pooled HPL (Aventacell HELIOS UltraGRO‐PURE GI GMP grade) at 5% CO_2_ and 37°C as described (Gimona et al. 2017; Pachler et al. 2017; Rohde et al. 2019). VSF1.02 containing UC‐MSC‐EVs was prepared from serum‐free (low glucose α‐MEM with Dipeptiven) conditioned medium after 24 h of incubation by tangential flow filtration (TFF) and diafiltration against Ringer's Lactate using a 100 kDa hollow fibre PES filter (Repligen). The resulting solution was sterile filtered through a 0.22 µm PES filter and immediately stored at −80°C.

The clinical grade EV‐suspension was prepared in a pharmaceutically certified manufacturing site that operates according to good manufacturing practice (GMP) at the Paracelsus Medical University, Salzburg, Austria. The GMP unit has obtained a European manufacturing and distribution licence for the human investigational medicinal product ‘MSC‐EV’, granted by the Austrian regulatory authorities. Rigorous and standardised characterisation of UC‐MSC‐EVs was performed as published previously (Warnecke et al. 2020) and complied with the criteria of the MISEV2018 guidelines (Thery et al. 2018), where applicable for therapeutic preparations (Martín‐Taboada et al. 2022). The ready‐to‐use suspension of UC‐MSC‐EVs was stored at −80°C and delivered to the operating room as a sterile solution in 2 mL vials. All clinical grade UC‐MSC‐EV batches were tested for the presence of endotoxins, bacterial sterility and the absence of mycoplasma. The identity, purity, potency and general safety parameters of the UC‐MSC‐EVs were characterised according to the established product release matrix of the GMP manufacturing unit at PMU Salzburg (Gimona et al. 2017; Pachler et al. 2017; Rohde et al. 2019). Quality control and potency parameters confirmed the previously published profile of UC‐MSC‐EVs.

Preparation of the batch VSF1.02 EV‐0050 that was used for injection contained 6.50 × 10^10^ particles/mL with a mean diameter of 130.7 nm as measured by nanoparticle tracking analysis (NTA, Model PMX110 from ParticleMetrix, Germany).

Total protein amount was determined using a QuBit 3.0 Fluorometer instrument (Life Technologies, CA, USA) and analyzed with the high sensitivity protein chip from Agilent according to the manufacturer´s instructions. Total protein amount of VSF1.02 batch EV‐0050 was 0.768 mg/mL amounting to 8.4 × 10^10^ particles/mg protein.

Surface profiling by multiplex flow cytometry (MACSPlex Exosome Kit, Miltenyi Biotec, Germany) demonstrated the presence of the tetraspanins CD9, CD63, CD81, as previously described (Warnecke et al. 2020) as well as MSC‐EV markers CD29, CD44, CD49e and CD73 and confirmed the absence of negative markers CD1/2/3/8/11c/14/19/20/24/25/31/40/45/56/69/86/133/142/209/326 and HLA‐ABC, HLA‐DR, ROR1, SSEA‐4.

Endogenous cytokine protein content was analysed using V‐Plex and U‐Plex human multiplex immunoassay kits on the Meso Scale Diagnostics platform according to the manufacturer's instructions, revealing absence of major pro‐inflammatory cyokines IL‐1ß, IL‐6, IL‐8, IL‐10 and TNF‐α.

Endotxin level was 0.8252 IU/mL.

The biological activity of this particular clinical grade EV preparation was confirmed through CD73 in vitro enzymatic assay as previously described (Priglinger et al. 2020). In brief, 10 µL of EVs (representing between 7 × 10^9^ and 3 × 10^10^ particles) in 10 mM HEPES (Sigma H3537) buffer containing 2 mM MgCl_2_ (Merck Millipore, MA, USA) were incubated with 10 µM AMP (Sigma 01930) for 20 min at 37°C. The amount of AMP consumption was detected with the AMP‐Glo Assay Kit (Promega, WI, USA) according to the manufacturer´s protocol and measured with Spark multimode microplate reader (Tecan, Austria). A total of 2 ng rhCD73 (Sigma N1665) was used as positive control and AMP‐CP (Sigma M8386) as CD73 inhibitor. The activity (displayed as AMP consumption) of the VSF1.02 batch EV‐0050 was at 1.10 × 10^−7^ µM/particle.

VSF1.02 was stored in 2 mL 2R injection vials (sterile made of Type 1 Clear glass, with Crimp neck 13 mm neck finish, pharmaceutical grade tubular glass, Dimensions (OD × H: 16 mm × 35 mm; Manufacturer: Nuova Ompi S.r.l. Via Molinella, 17, 35017 Piombino Dese, Italy).

## 3 Discussion

To the best of our knowledge, we here report the first in‐human application of UC‐MSC‐EVs in a patient with spina bifida to test the technical feasibility of local administration. In addition, their potential to ameliorate intrathecal fibrosis and neurodegeneration after tethered cord release surgery should be investigated. Despite the promising result observed, several challenges and limitations must be addressed before the technique can be widely adopted.

### 3.1 Technical Feasibility and Dosage

While the intraoperative application of UC‐MSC‐EVs was technically feasible in this case, determining the optimal dosage, frequency, and method of delivery remains a challenge. The dosage used was based on prior experimental studies and was limited to a single application during surgery. It is unclear whether multiple applications via alternative routes or higher doses could yield better outcomes, or if there are risks of overdosing, such as adverse immune reactions or excessive fibrosis (Martín‐Taboada et al. 2022). Additional research in animal models and early‐phase clinical trials will be necessary to elucidate these aspects.

### 3.2 Long‐Term Safety

One of the primary limitations of this study is the lack of large cohort long‐term follow‐up data to assess the enduring effects of UC‐MSC‐EV therapy. While the short‐term outcomes in this case were positive, including reduced syringomyelia and oedema and stable neurological function, the potential for delayed adverse effects or recurrence of fibrosis and tethering remains. Since fibrosis and scarring are processes that evolve over time, long‐term monitoring is critical to understanding whether UC‐MSC‐EVs can offer sustained positive clinical courses. Moreover, long‐term outcomes need to be compared to patients receiving standard surgical care without EV therapy to establish the true efficacy of this approach in carefully designed clinical studies.

### 3.3 Lack of Control Group and Randomisation

This case report describes an individual healing attempt. Thus, it lacks control group and randomisation. While promising, it is impossible to draw any definitive conclusion about the benefits of UC‐MSC‐EVs compared to standard therapy. Future studies, particularly randomised controlled clinical trials, are mandatory to rigorously assess whether UC‐MSC‐EVs significantly improve neurological outcomes compared to conventional treatments or other emerging therapies.

### 3.4 Comparison With Existing Therapies

Currently, no therapeutic interventions exist to specifically prevent fibrosis and scarring in spina bifida surgeries, other than meticulous surgical techniques aimed at minimising trauma to neural structures. Yet ONTD patients harbour a life‐long risk of neurological deterioration after surgical repair because of intrathecal fibrosis and scarring resulting in a tethered cord syndrome (Almadori and Butler 2024; Borgstedt‐Bakke et al. 2020; Brock et al. 2019; Brun et al. 2023; Houtrow et al. 2021; Inversetti et al. 2019; Johnson et al. 2023; Sileo et al. 2019; Spoor et al. 2023; Weaver et al. 2023). Recent follow‐up analysis have quoted the incidence of symptomatic tethered cord and subsequent neurosurgical procedures as high as 27% in prenatal and 15% in postnatal MOMS cohort (Inversetti et al. 2019; Paslaru et al. 2021; Spoor et al. 2023). Also epidermoid cysts within the placode were detected more often in prenatal than the postnatal group (11% vs. 3%, resp.). In a Danish population study, 45 out of 166 patients with ONTD who underwent untethering, the most common indications were progressive spine deformity (40%), deteriorating ambulation (38%), and deteriorating neurogenic bladder and/or bowel dysfunction (32%) (Borgstedt‐Bakke et al. 2020).

Additionally, tethered cord release procedures also bear a 15% risk of re‐tethering due to post‐operative arachnoid scarring (Borgstedt‐Bakke et al. 2020; Paslaru et al. 2021). Thus, the significant decline of ambulatory ability in prenatal cohorts, the high rates of tethered cord syndrome at early ages still pose a controversy about the best treatment strategy (Inversetti et al. 2019; Paslaru et al. 2021; Sileo et al. 2019; Spoor et al. 2023; Weaver et al. 2023).

Consequently, any measure that is able to minimise post‐operative fibrosis and intrathecal scarring as well as any neuroprotective agent that can reduce the direct damage to neural structure, appears outstandingly helpful for neurosurgical interventions in spina bifida patients.

MSC‐EVs act in a mainly paracrine manner to mediate and stimulate cellular processes important for tissue repair, regeneration and immune cell modulation (Gomzikova et al. 2019). Neuro‐regenerative and immunosuppressive activities (Doeppner et al. 2015) as well as activities that attenuate neuro‐inflammation and scarring after spinal cord injury (Romanelli et al. 2019) have been described (Doeppner et al. 2015; Romanelli et al. 2019). We have also shown that our human‐derived UC‐MSC‐EVs exert immunomodulatory activity on microglial cells (Warnecke et al. 2020) and improve the survival of spiral ganglion neuron in vitro (Warnecke et al. 2020). Moreover, we have demonstrated that local application of UC‐MSC‐EVs attenuated hearing loss and protect auditory hair cells from noise‐induced trauma in a clinically relevant mouse model. Finally, clinical data from an individual healing attempt (Warnecke et al. 2021) with the aim to reduce cochlea implant‐related inflammation and fibrosis in the inner ear confirmed a satisfactory safety profile for topical application of UC‐MSC‐EVs in this particular patient (Warnecke et al. 2021). It joins the line with other first‐in‐human applications, for example, for wound healing (Johnson et al. 2023). However, the mechanisms of action exerted by the UC‐MSC‐EVs in spina surgery need to be further investigated to be clarified.

### 3.5 Immunogenicity and Heterogeneity of Response

Although no adverse immune responses were observed in this patient, there remains a theoretical risk of immunogenicity or variability in response to UC‐MSC‐EVs across different patient populations (Martín‐Taboada et al. 2022). Manufacturing‐related factors (such as the source and passage number of the mesenchymal stromal cells, and the method of vesicle isolation), and patient‐specific factors (e.g., immune status, underlying conditions) could all influence variability and the therapeutic potency of the substance and how well patients respond to this therapy, respectively. Further research is needed to understand the variability in treatment outcomes and to mitigate potential risks.

## 4 Conclusion

Direct application of UC‐MSC‐EVs onto the neural structures during spina bifida surgery appears to have potential for further larger scale evaluation of therapeutic effects—both in preclinical animal models of ONTD and Phase I/II clinical trials for safety and efficacy. However, whether local EV application can lead to significant long‐term neurological benefits needs to be studied thoroughly in randomised controlled trials.

## Author Contributions

**Matthias Krause**: Conceptualization (equal); investigation (equal); resources (equal); writing–original draft (equal); writing–review and editing (equal). **Janina Gburek‐Augustat**: Formal analysis (equal); investigation (equal); resources (equal); writing–review and editing (equal). **Daniel Gräfe**: Investigation (equal); resources (equal); writing–review and editing (equal). **Roman Metzger**: Project administration (equal); validation (equal); writing–review and editing (equal). **Marco Ginzel**: Formal analysis (equal); validation (equal); writing–review and editing (equal). **Christoph J. Griessenauer**: Investigation (equal); supervision (equal); writing–review and editing (equal). **Lukas Grassner**: Formal analysis (equal); writing–review and editing (equal). **Daniel Weghuber**: Conceptualization (equal); funding acquisition (equal); supervision (equal); writing–review and editing (equal). **Johann Gradl**: Data curation (equal); visualization (equal); writing–review and editing (equal). **Daniela Auer**: Methodology (equal); resources (equal); writing–review and editing (equal). **Tanja Schally**: Methodology (equal); resources (equal); writing–review and editing (equal). **Stefan Rund**: Methodology (equal); resources (equal); writing–review and editing (equal). **Carina Kals**: Methodology (equal); resources (equal); writing–review and editing (equal). **Christina Folie**: Methodology (equal); resources (equal); writing–review and editing (equal). **Elisabeth Bayer**: Methodology (equal); resources (equal); writing–review and editing (equal). **Mario Gimona**: Conceptualization (equal); methodology (equal); project administration (equal); resources (equal); supervision (equal); validation (equal); writing–review and editing (equal). **Eva Rohde**: Conceptualization (equal); methodology (equal); project administration (equal); supervision (equal); writing–review and editing (equal).

## Ethics Statement

The single named patient use was reviewed by the internal review board of the University Hospital Salzburg. The patient is registered to the SBHC Registry study (IRB #1062/2024 at Ethikkommission des Landes Salzburg).

## Conflicts of Interest

The authors declare no conflicts of interest.

## Data Availability

The data that support the findings of this study are available on request from the corresponding author. The data are not publicly available due to privacy or ethical restrictions.
